# Relationships Among In-Line Milk Fat-to-Protein Ratio, Metabolic Profile, and Inflammatory Biomarkers During Early Stage of Lactation in Dairy Cows

**DOI:** 10.3390/vetsci12020187

**Published:** 2025-02-19

**Authors:** Karina Džermeikaitė, Justina Krištolaitytė, Neringa Sutkevičienė, Toma Vilkonienė, Gintarė Vaičiulienė, Audronė Rekešiūtė, Akvilė Girdauskaitė, Samanta Arlauskaitė, Árpád Csaba Bajcsy, Ramūnas Antanaitis

**Affiliations:** 1 Large Animal Clinic, Veterinary Academy, Lithuania University of Health Sciences, Tilžės Str. 18, LT-47181 Kaunas, Lithuania; justina.kristolaityte@lsmu.lt (J.K.); neringa.sutkeviciene@lsmu.lt (N.S.); toma.vilkoniene@lsmu.lt (T.V.); gintare.vaiciuliene@lsmu.lt (G.V.); audrone.rekesiute@lsmu.lt (A.R.); akvile.girdauskaite@lsmu.lt (A.G.); samanta.arlauskaite@lsmu.lt (S.A.); ramunas.antanaitis@lsmu.lt (R.A.); 2 Clinic for Cattle, University of Veterinary Medicine Hannover, Foundation, Bischofsholer Damm 15, 30173 Hannover, Germany; csaba.bajcsy@tiho-hannover.de

**Keywords:** biomarkers, transition period, negative energy balance, herd health, acute-phase protein

## Abstract

**Simple Summary:**

This study examined the relationships among blood nonesterified fatty acids (NEFAs), serum amyloid A (SAA), milk composition, and dairy cattle health. A significant positive association was discovered between NEFAs and milk fat content (r = 0.459, *p* < 0.001) as well as the milk fat-to-protein ratio (r = 0.516, *p* < 0.001). Cows in the high NEFA (II-NEFA) class had higher milk fat content and milk fat-to-protein ratios compared to those in the low NEFA (I-NEFA) class (4.20% vs. 3.81% and 1.33 vs. 1.17, respectively). These findings indicate that elevated NEFA levels promote milk fat synthesis, likely due to fat mobilisation associated with negative energy balance (NEB). Significant negative correlations were identified between SAA levels and both milk fat content (r = −0.426, *p* < 0.001) and the milk fat-to-protein ratio (r = −0.535, *p* < 0.001), suggesting that inflammation may inhibit milk fat production. Furthermore, elevated SAA levels were positively associated with increased cow activity (r = 0.382, *p* < 0.001), indicating potential discomfort-driven behavioural changes related to inflammation. Milk composition reflects the metabolic and inflammatory conditions in dairy cows, suggesting that it could be used to measure energy balance and health, thereby avoiding blood collection and analysis. During early lactation, NEB elevates NEFA levels, which may lead to metabolic disorders such as fatty liver syndrome and ketosis. High NEFA levels also compromise immune function, increasing the susceptibility to conditions like mastitis. Monitoring milk components could enable the early detection of NEB and inflammatory risks, facilitating timely interventions to improve cow health and productivity. Additionally, tracking SAA levels and using automated behavioural monitoring systems may enhance dairy farm management by promoting better health, production, and welfare outcomes. However, further research is required to fully understand the interplay among NEFAs, inflammation, and metabolic disorders in dairy cows.

**Abstract:**

The early lactation phase in dairy cows is characterised by significant metabolic and inflammatory changes. This study aimed to evaluate the relationship between serum nonesterified fatty acids (NEFAs), a marker of negative energy balance (NEB), and serum amyloid A (SAA), an indicator of systemic inflammation. Blood samples were collected from 71 Holstein cows during the transition period 17 (±3) DIM, and serum concentrations of NEFAs and SAA were measured. The results revealed a significant negative correlation between NEFAs and SAA (r = −0.441, *p* < 0.001), suggesting that increased fat mobilisation may suppress the inflammatory response, thereby increasing the susceptibility to metabolic and infectious diseases. The emerging research indicates a negative association between SAA levels and milk fat-to-protein ratio in dairy cows, particularly under inflammatory conditions. The research indicates that elevated levels of SAA, which is an inflammatory biomarker, are frequently associated with alterations in milk composition, including a reduced fat-to-protein ratio. This study examined the correlations among serum NEFAs, SAA, milk composition, and dairy cattle health. A strong positive correlation was identified between serum NEFAs and milk fat content (r = 0.459, *p* < 0.001), as well as between serum NEFAs and the milk fat-to-protein ratio (r = 0.516, *p* < 0.001). Cows with elevated serum NEFA levels (classified as II-NEFA) exhibited significantly higher milk fat content (4.20%) and milk fat-to-protein ratios (1.33) compared to cows with lower serum NEFA levels (I-NEFA class; 3.81% and 1.17, respectively). The data indicate that elevated serum NEFA levels are associated with an increased milk fat synthesis, likely driven by enhanced fat mobilisation during NEB. A significant negative correlation was observed between SAA and both milk fat content (r = −0.426, *p* < 0.001) and the milk fat-to-protein ratio (r = −0.535, *p* < 0.001), indicating that inflammation may impair milk fat production. Elevated SAA levels were also associated with increased cow activity (r = 0.382, *p* < 0.001), suggesting that inflammation may lead to behavioural changes driven by discomfort. Our findings suggest that milk composition reflects the metabolic and inflammatory status of dairy cows and could serve as a non-invasive alternative to blood sampling for assessing energy balance and health. NEB, which typifies early lactation, promotes fat mobilisation, resulting in elevated serum NEFA levels and an increased risk of metabolic disorders such as fatty liver syndrome and ketosis. Moreover, high serum NEFA levels adversely affect immune function, increasing vulnerability to infections such as mastitis. Monitoring milk composition may enable the early detection of NEB and inflammatory conditions, thereby supporting proactive health management. However, further research is necessary to elucidate the role of NEFAs and inflammation in the development of metabolic diseases in cattle.

## 1. Introduction

During the onset of lactation, especially high producing dairy cows are more prone to experience a higher occurrence of diseases [1]. These can negatively affect fertility, milk yield, and subsequent lactation. Furthermore, early postpartum negative energy balance (NEB) is also linked to a higher occurrence of viral diseases and metabolic disorders [2].

Understanding the metabolic alterations in dairy cows, as well as their diagnosis and detection, is of utmost significance [3] for improving cattle health and welfare through appropriate nutrition and management strategies [4]. The relationship between energy balance in dairy cows and metabolic blood markers (such as β-hydroxybutyrate acid (BHB), aspartate aminotransferase, and gamma-glutamyl transferase activities) and milk composition has been well documented [5]. Significant metabolic changes occur during the transition period (three weeks prepartum) and early lactation (three weeks postpartum), contributing substantially to health issues in dairy cows. These changes are characterised by profound alterations in dietary metabolism, endocrine function, and immune responses [6,7,8]. This stage is marked by an increased need for nutrients for foetal growth, colostrum production and nursing of the newborn, and milk production. The parameters of energy metabolism, such as nonesterified fatty acids (NEFAs), are indicative of these metabolic shifts, with prepartum serum concentrations being lower than those observed postpartum [9]. In addition, abrupt and profound physiological and metabolic transformations occur postpartum, often resulting in behavioural changes. During this phase, cows must adapt to the demands of lactation, undergoing at least five major physiological processes: (1) diminished immune competence, (2) adverse energy balance, (3) hypocalcaemia, (4) inflammatory reactions, and (5) oxidative stress. The prolonged or severe disruption of these processes can lead to metabolic and infectious diseases as well as endocrine dysfunction [10].

Metabolic profiles are valuable tools for forecasting or identifying potential disorders at an early stage [9]. One approach to assessing the metabolic health and nutritional status of dairy cows is through metabolic profile testing [11]. Blood composition analysis is among the most effective methods for diagnosing metabolic and nutritional imbalances in cows. Various blood tests can measure parameters such as NEFAs, BHB, and substances that support liver function [5]. Quantifying the levels of NEFAs and BHB in the blood (serum or plasma) is widely regarded as one of the most reliable methods for evaluating an organism’s metabolic well-being [12]. Excessive lipid mobilisation, particularly during early lactation, to meet energy demands leads to metabolic stress. This is characterised by elevated levels of NEFAs [13], followed by increased BHB concentrations and reduced levels of blood glucose [14]. Additionally, changes in the milk fat-to-protein ratio (F:P), an increase in triacylglycerol content, and fatty infiltration of the liver may occur [15].

The natural immune system employs a wide range of defence mechanisms, among which acute-phase proteins (APPs) hold particular significance [16]. The acute-phase reaction is a non-specific immune response, typically triggered by inflammatory cytokines, and involves the production of APPs. These proteins are either absent or present only in minimal amounts in healthy animals. Serum amyloid A (SAA) is considered the primary acute-phase protein in various animals, including both domestic and wild ruminants. In cows, SAA concentrations increase significantly during the peripartal period. During pregnancy, levels of SAA and C-reactive protein (CRP) initially rise, peaking between 60 and 120 days, before gradually declining toward the latter stages of pregnancy (beyond 180 days). This increase is potentially linked to inflammatory conditions caused by stress or lesions in the genital tract. The rise in APPs after calving may be attributable to factors associated with colostrum production and/or the calving process, rather than to disease-related processes. SAA and other APPs have been shown to be present in lower concentrations in healthy cows’ mammary glands, colostrum, plasma, and milk [17].

For effective strategies, the routine identification of biomarkers that accurately characterise an animal’s physiological status is essential [18]. While metabolic profile testing offers numerous benefits, regularly testing the blood of animals involves significant costs, logistical challenges, and invasiveness. This has led to the extensive investigation of milk as a biofluid for monitoring the metabolic status and overall health of dairy cows, owing to its readily available nature [11]. The potential to predict blood metabolites from milk samples collected during routine milking has gained considerable attention [19]. Milk composition is known to vary throughout lactation, with the most significant changes occurring during the early and late stages of lactation [20]. Indicators such as the milk fat-to-protein ratio, lactose content, and somatic cell count (SCC) serve as important markers of energy balance in dairy cows [21]. Additionally, changes in behaviours such as feeding and rumination duration may signal underlying issues with the animal’s welfare and comfort [22].

Monitoring the temperature and pH levels in the reticulorumen of fresh dairy cows in line enables the assessment of the potential for subacute ruminal acidosis (SARA) and offers the chance to determine the preventive impact of these devices [23]. Continuous monitoring of the reticulorumen pH is now possible with commercially available boluses that are introduced orally and placed in the reticulum [24]. The SmaXtec (SmaXtec Animal Care Technology^®^, Graz, Austria) system is an additional cutting-edge technology employed in dairy farms to evaluate the well-being of cows. SmaXtec enables continuous in-line monitoring of data, including ruminal pH temperature and activity.

Scientific research has demonstrated that serum APP concentrations alter during calving [17,25,26,27]. Nevertheless, according to our previous results, no sufficient evidence exists regarding the correlation among SAA and blood metabolic profiles, milk quality, and cows’ behaviour throughout early lactation. However, to our knowledge, there is a lack of research examining the relationship between APP and milk parameters. This research gap presents an opportunity to explore how changes in acute-phase proteins may impact the overall health and productivity of dairy cows during early lactation. Thus, our hypothesis and goals in this investigation are as follows.

The aim of our study, based on our hypothesis, is to evaluate the kinetics of changes in the SAA levels, metabolic profile, milk quality, and cows’ behaviour during early lactation in healthy dairy cows.

Our hypothesis posits that cows experiencing metabolic imbalances at the onset of lactation are more prone to developing SAA levels during this period. We anticipate that variations in SAA levels will reflect this susceptibility.

## 2. Materials and Methods

### 2.1. Location of the Farm

This study followed the requirements outlined in the Lithuanian Law on Animal Welfare and Protection, which has the approval number PK012858. The experiment commenced on 1 June 2024 and concluded on 1 August 2024. It was conducted on a Lithuanian dairy farm housing 1500 milking cows in free-stall barns. The coordinates of the farm are 54.97378759003201 latitude and 23.76954146935687 longitude. The producer employs a DeLaval milking parlour (DeLaval Inc., Tumba, Sweden) to milk 1000 of the 1500 cows twice daily. The free-stall barns, equipped with ventilation systems (DeLaval Inc., Tumba, Sweden), provided housing for the cows. Milking was carried out in the parlour system twice daily, at 5 a.m. and 5 p.m. For this study, a total of 71 milking cows were selected from a sample of 1000 cows that underwent clinical examinations. These cows were chosen specifically from their second or subsequent lactations, within 17 (±3) days post-calving. Only healthy animals were included, based on comprehensive clinical assessments. Each cow underwent a thorough physical examination to confirm its overall health and to ensure the absence of systemic illnesses or debilitating conditions. The evaluation involved assessing the cows’ general appearance and behaviour for any signs of illness. Cows exhibiting signs of systemic illness were excluded to ensure data reliability and minimise variability. The clinical examinations confirmed that all selected cows were healthy, with no clinical evidence of illness. During the study, 71 blood samples were collected from cows. The investigation period spanned from calving to 100 days in milk (DIM), encompassing the early stage of lactation. During this period, the energy demands of intensive milk production significantly increase, rendering dairy cows highly susceptible to NEB [28]. Dairy cows typically experience such a negative energy status during the early stages of lactation. The developing NEB triggers the release of stored fat to make up for the reduced food consumption, resulting in elevated NEFAs, which in certain instances may lead to the formation of BHB, which appears in the blood [29]. The mean weight of the selected cows was 550 kg ± 45 kg, with an average energy-corrected milk yield (4.2% fat and 3.6% protein) of 12,500 kg per cow per lactation. Calculations were performed using established formulas [30].

The breed, lactation number, last calving date, and milk yield were retrieved from the farm’s computer system (Delpro, DeLaval Inc., Tumba, Sweden) and recorded in a spreadsheet. The number of DIM for each cow was calculated for each data collection period by determining the number of days between the last calving date and the first day of the data collection period. The experimental dairy cows were fed a total mixed ration (TMR) tailored to meet their physiological requirements. Feeding took place twice daily, at 6 a.m. and 6 p.m., and the cows had unrestricted access to water. The feed was meticulously formulated by a nutritionist to ensure that the cows received all essential nutrients necessary for optimal health and milk production. Any leftover feed was removed daily at 5 a.m. and 5 p.m. Table 1 presents the nutritional composition of the diet.

### 2.2. Collected Variables

The BROLIS HerdLine in-line milk analyser (Brolis Sensor Technology, Vilnius, Lithuania) was utilised to measure milk composition in this study. Behavioural parameters of the cows, including rumination time (min./day), body temperature (°C), reticulorumen pH, water consumption (L/day), and activity (h/day), were assessed using SmaXtec boluses (SmaXtec Animal Care GmbH, Graz, Austria). Additionally, serum NEFA and SAA levels were measured. By integrating these technologies, we collected a wide range of data on the health and performance of the dairy cows. The data collected from the Brolis Sensor Technology and SmaXtec boluses enabled us to monitor the cows’ health status and well-being in real time. The combination of rumination time, body temperature, pH levels, water consumption, activity levels, and blood parameters provided a comprehensive overview of the cows’ overall condition.

### 2.3. Blood Parameters

Blood samples were collected from each cow four hours after morning milking and feeding. All samples were taken during the clinical examination. The cows were restrained in a resting stall or headlock during each sampling to obtain a small blood sample from the coccygeal vein using a needle syringe. The blood samples were collected from the coccygeal vein using an evacuated tube without anticoagulant (BD Vacutainer^®^, Eysin, Switzerland) for the purpose of analysing the biochemical profile of the blood. Within one hour of collection, the blood samples were transported at a temperature of +4 °C to the Laboratory of Clinical Tests at the Large Animal Clinic of the Veterinary Academy, Lithuanian University of Health Sciences, for further analysis. Subsequently, in the laboratory, blood samples were centrifugated for 15 min at 1500× *g*.

NEFA concentrations were assessed using an automated wet chemistry analyser (Rx Daytona, Randox Laboratories Ltd., London, UK). Selectra Junior (Vital Scientific, Dieren, The Netherlands) ELISA was used to measure SAA concentrations.

### 2.4. Milk Parameters

During this experiment, the milk composition was documented utilising the BROLIS HerdLine in-line milk analyser. This equipment was utilised to continually measure milk fat, protein, and F:P from every milk sample. The device was equipped with a GaSb broadly tunable external cavity laser-based spectrometer that operates in the 2100–2400 nm spectral range. It monitors milk flow in transmission mode throughout the milking process, enabling individual measurements. By analysing molecular absorption spectra, it effectively functions as a compact, on-farm laboratory, determining the concentrations of the primary components in the milk. This compact “mini-spectroscope” is conveniently located in the milking parlour and attached along the milk line.

In the Eurofins laboratory, the accuracy of each BROLIS HerdLine in-line milk analyser was assessed and calibrated. The root mean square error of prediction (RMSEP) values for fat, protein, and lactose were 0.21%, 0.19%, and 0.19%, respectively.

### 2.5. Cow Behaviour

**Figure 1:**
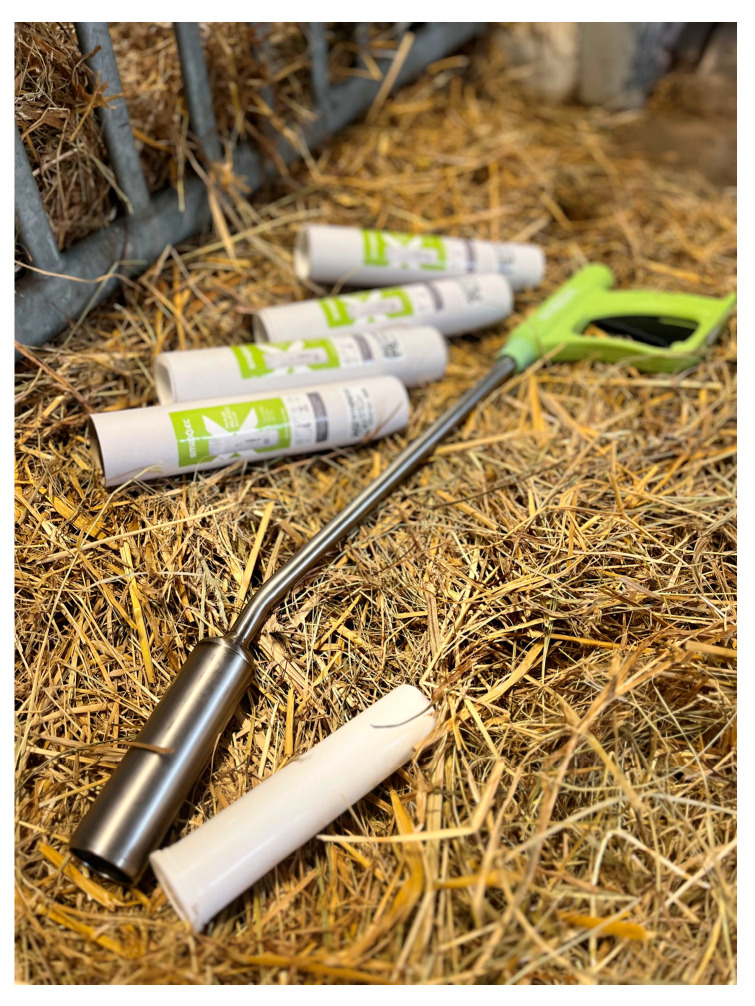
SmaXtec boluses and special introducer device.

At the beginning of the trial, each of the 71 cows received an orally applicated SmaXtec bolus within the first 30 days after their calving. The boluses were introduced into the reticulorumen using a specialised applicator device, following the manufacturer’s instructions. Prior to application, each bolus was activated and cross-referenced with the ear tag number of the respective cow (Figure 1). A connection was also established with the base station. The SmaXtec boluses recorded the changes in various parameters, such as reticulorumen pH, temperature, and walking activity, enabling the in-line monitoring of this data. Initially, pH calibration was performed using buffer solutions with pH values of 4 and 7, provided by Reagecon (Shannon, Ireland). The data were recorded at ten-minute intervals daily. The SmaXtec Messenger^®^ software compiled and presented all the gathered information.

SmaXtec utilises bolus technology to collect data on the drinking behaviour of individual cows by directly monitoring the reticulum. The bolus device tracks the animal’s internal body temperature and estimates the quantity of water consumed by employing AI-driven algorithms that analyse temperature fluctuations following each drinking event. This enables the monitoring of each cow’s water intake to ensure that it meets the appropriate thresholds without requiring additional effort. The data are then transmitted wirelessly to a central system, where it can be analysed by farmers or veterinarians. This technology offers a non-invasive and efficient method for safeguarding the well-being of individual animals within the herd, ultimately enhancing overall herd health and productivity.

An implanted and wireless device was utilised to record the reticulorumen temperature (RT), pH, total reticulated rumination (TRR), and physical activity. The data acquisition was facilitated by antennas from SmaXtec Animal Care Technology^®^. The microprocessor-managed system captured pH and TRR data via an analogue-to-digital (A/D) converter and stored it on an external memory chip for subsequent analysis. The collected was compiled by the SmaXtec Messenger^®^ software (Version 4). This innovative technology enabled in-line monitoring of key indicators of the animals’ health and behaviour, allowing for the early detection of any potential issues.

### 2.6. Group Formation

All blood samples from cows (*n* = 71) were divided into two groups based on NEFA concentrations: group I-NEFA had a NEFA concentration of 0.24 ± 0.12 mmol/L (*n* = 43), while group II-NEFA had a concentration of 0.87 ± 0.23 mmol/L (*n* = 28). The research of Tessari et al. served as the basis for choosing the NEFA threshold as the cut-off value [31]. This value indicates excessive lipomobilisation and may serve as a criterion for monitoring dairy cows at risk of postpartum illnesses.

### 2.7. Statistical Analysis

The statistical analysis was performed using the SPSS 26.0 (SPSS Inc., Chicago, IL, USA) package. Student’s *t*-test and analysis of variance (ANOVA) was applied to compare the average values of the I-NEFA and II-NEFA class, which were normally distributed. The correlation coefficients were calculated to determine the relationships among various blood biochemical parameters, enzymes, physiological indicators (SmaXtec parameters), and milk composition. The Pearson correlation was used to detect the linear relationship between the investigated traits.

## 3. Results

### 3.1. Correlation Among Blood, Milk, and Behaviour Parameters

We found a significant negative association between blood NEFAs and SAA (r = −0.441, *p* < 0.001). As NEFAs increase, blood SAA decreases. We found strong positive correlations between NEFAs and milk fat (r = 0.459, *p* < 0.001) and NEFAs and milk fat-to-protein ratio (r = 0.516, *p* < 0.001) (Table 2).

We found a strong positive correlation between SAA and cow activity (r = 0.382, *p* < 0.001). A strong negative correlation was found between SAA and milk fat-to-protein ratio (r = −0.535, *p* < 0.001) and SAA and milk fat (r = −0.426, *p* < 0.001).

### 3.2. Impact of NEFA Classifications on Milk Characteristics

We found significant differences in milk fat between cows in the I-NEFA class (NEFA 0.24 ± 0.12) and those in the II-NEFA class (NEFA 0.87 ± 0.23). The milk fat in the I-NEFA class of cows was 3.81%, while in the II-NEFA class, it was 4.20%. The milk fat content in the II-NEFA class exceeded that of the I-NEFA class by 10.24%. Analysing the milk F:P revealed significant distinctions among NEFA classes. The milk F:P in the I-NEFA class of cows was 1.17, but in the II-NEFA class, it was 1.33. The F:P in milk was 13.67% greater in the class II-NEFA than in the I-NEFA class (Table 3). These findings suggest that there is a clear relationship between NEFA levels and milk composition, specifically in terms of fat content and F:P.

According to our results, we did not find any significant differences (*p* > 0.05) between the I-NEFA and II-NEFA class cows in their milk protein.

### 3.3. Impact of NEFA Classifications on the Blood Parameters

We found significant differences in cow SAA between the I-NEFA class (NEFA 0.24 ± 0.12) and those at the II-NEFA class (NEFA 0.87 ± 0.23). The SAA in the I-NEFA class of cows was 77.84 μg/mL, while in the II-NEFA class, it was 59.01 μg/mL. It was a 24.19% reduced concentration in the II-NEFA class cows compared to the I-NEFA class cows (Table 4).

## 4. Discussion

The results indicate that cows in the II-NEFA class had higher milk fat content compared to those in the I-NEFA class. These results showed that with increasing serum NEFA concentrations, milk fat content and milk F:P also increase. This indicates a direct relationship between serum NEFAs and milk quality. As NEFA levels increase, milk fat and the F:P also increase, indicating a potential link between lipid metabolism and milk production. Elevated NEFA levels suggest an increased fat mobilisation from body stores, leading to a greater availability of fatty acids for milk fat synthesis. This relationship is commonly observed in dairy cows during metabolic stress, particularly in early lactation when they mobilise body fat to support milk production. Milk components have the potential to indicate cows at risk of health problems and NEB, especially during early lactation. Monitoring milk components provides valuable insights into the nutritional status and overall health of dairy cows, enabling early intervention and prevention of potential issues [32]. During the early lactation phase, the increased energy demands for milk production, combined with a comparatively low DMI, can lead to an energy deficit, or NEB [33]. According to the research, the F:P ratio is highest during the early lactation phase when the energy deficit is most significant, and the energy balance stabilises once the decline in the F:P ceases [34]. To meet these energy demands, body fat and protein are mobilised for hepatic gluconeogenesis, resulting in elevated plasma levels of NEFAs, BHB, and ammonia [35]. In case of a severe energy demand, excessive NEFA production appears in the bloodstream, which the liver absorbs. The synthesis of triacylglycerols becomes intensive, potentially leading to fatty liver syndrome and ketosis. Moreover, elevated NEFA levels can negatively impact immune system functionality, increasing the susceptibility to infections such as mastitis and metritis [32]. Blood sampling is expensive and impractical for routine early detection of health issues, despite yielding reliable data [18]. Consequently, in recent years, alternative physiological samples, such as milk, have been explored for energy balance estimates due to their ease of collection and availability [32]. Both fat and protein levels in milk serve as indicators of the animals’ energy situation. During energy-deficient phases, such as early and later lactation, the milk fat content is typically elevated [36]. During lactation, the mammary gland’s demand for glucose leads to an increased gluconeogenesis and glycogenolysis in the liver; lipolysis in adipose tissue, which results in the release of NEFAs into the bloodstream; and the mobilisation of labile protein from muscle tissue. These elevated demands result in significant alterations in lipid, protein, and mineral metabolism, with the liver and adipose tissues playing key roles. The mobilisation of lipids and proteins causes weight loss, leading to a reduction in the body condition score [15]. The activation of lipolysis in NEB results in increased quantities of NEFAs in the blood, hence promoting fat oxidation. The saturation of the liver is linked to the production of BHB, which causes high levels in both blood and milk [37]. The research indicates that relationships between breeding values for milk BHB and commonly assessed qualities suggest that selecting for reduced milk BHB during early lactation will enhance health and fertility. A reduced milk BHB was genetically correlated with a diminished F:P, an elevated body condition score, and a decreased incidence of clinical ketosis [38]. The liver absorbs NEFAs from the plasma, which is esterified to form triacylglycerol (TAG) [39]. When the liver cannot handle the excess NEFAs that enters, TAG starts accumulating in hepatocytes, leading to hepatic steatosis and changes in the size of the liver and other structures, such as the portal vein. A negative energy balance and the subsequent drop in blood glucose levels trigger fat mobilisation, leading to the production of NEFAs, BHB, acetoacetate, and acetone [15]. The corresponding rise in the milk F:P is closely linked to the stages of NEB and fat tissue mobilisation. A milk F:P of 1.35 to 1.50 may indicate energy insufficiency. A lower milk F:P may suggest SARA due to a decreased production of rumen volatile fatty acids (acetate and butyrate), which are precursors for mammary fatty acid synthesis [18]. Based on our results, estimating the energy balance from milk constituents offers an alternative, as milk samples are easily obtained and can be monitored daily. Further investigation into the relationship between changes in milk F:P and specific metabolic processes in dairy cows could lead to advancements in optimizing feeding strategies and maximizing milk production efficiency.

A moderate positive correlation was identified between blood SAA levels and cow activity (r = 0.382, *p* < 0.001). This indicates that elevated blood SAA levels correlate with higher cow activity. This increased activity can be attributed to the cows experiencing discomfort or pain due to inflammation. The inflammatory response to infection is the primary factor driving the alterations in behaviour [40]. Several substances, including eicosanoids and cytokines, like inflammatory factors, cause inflammation. The most effective triggers of the inflammatory response are these mediators, which activated accessory immune cells (such as macrophages and monocytes) produced at the site of infection. Cytokines are responsible for inducing the acute-phase response, which is brief and only lasts a few hours [41]. Inflammatory conditions often cause pain and stress, which can trigger behavioural changes, leading to increased movement as cows seek to alleviate symptoms or find relief. Based on the research, cows with clinical mastitis decreased their time spent reclining and increased the frequency of lying bouts and stepping. The alterations in activity patterns stem from the discomfort cows endure while lying down owing to engorged udders and their dissatisfaction from insufficient rest time [42]. The literature, which regards increases in SAA as a hallmark of the acute-phase response to inflammation and a sensitive indicator of health disturbances in cows, supports this. Huzzey et al. (2007) found that cows with clinical mastitis demonstrated considerable increases in activity, as shown by more frequent laying, likely due to the pain resulting from udder inflammation [43]. For instance, cows with clinical mastitis from *Escherichia coli* endotoxin preferred to stand and avoided lying on the affected side [44]. Based on a recent study, it was found that the cows were more restless during milking and had a lowered lying time, contrary to what is normally seen in sick animals [40]. This is consistent with the findings of our study, that the activity of cows can increase in cases of inflammation (serum SAA increases). The behavioural adaptations seen in cows with elevated SAA levels are responses to the discomfort and pain associated with inflammation. These changes in activity patterns can be used in conjunction with SAA monitoring to improve the detection of health disorders, allowing for proactive management and better animal welfare outcomes. As the research advances, integrating SAA level monitoring with automated behavioural tracking systems could further enhance the precision of health assessments in dairy farming, leading to improved productivity and sustainability in the industry. Monitoring SAA levels in cows can help farmers detect and address potential health issues before they escalate, ultimately leading to improved herd management and productivity.

A strong negative association between milk F:P and SAA was observed (r = −0.535, *p* < 0.001), as well as between SAA and milk fat (r = −0.426, *p* < 0.001). These data indicate that an increase in blood SAA levels correlates with a decrease in both milk F:P and milk fat levels. This suggests a possible correlation between inflammation and alterations in milk composition. Ruminal acidosis is clinically correlated with reduced dry matter intake and diminished milk fat, frequently occurring in conjunction with transition period metabolic disorders such as fatty liver, ketosis, and hypocalcaemia, indicating potential associations among acidosis, inflammation, and metabolic diseases in cattle [45]. SARA has long been presumed to induce milk fat depression [46]. The recent research indicates that cows subjected to an experimental fast transition to a high-grain diet (75% concentrates) and a change in housing arrangement exhibited an increase in SAA as positive-phase proteins [47]. Indeed, SARA leads to the intensive synthesis of SAA and, in certain instances, haptoglobin (Hp). Junfei Guo et al. reported that interleukin-1 (IL-1) and SAA levels in the liver were markedly elevated in SARA cows [48]. The presence of pathogens and lipopolysaccharide (LPS) in the bloodstream triggers inflammatory responses. Plasma LPS induces a systemic acute-phase response (APR) that is linked to pro-inflammatory cytokines in the liver. LPS-binding protein (LBP) is a specific marker that rises a lot during SARA. This makes it easier for LPS to attach to membrane-associated receptors, which boosts the immune response. Along with LBP, LPS turns on nuclear factor-kappa B (NF-κB). This then turns on the pro-inflammatory mediators of APR, such as IL-1β, IL-6, and TNF-α. These are made by macrophages or blood monocytes at the site of the injury or infection [48]. These cytokines are anticipated to stimulate the production of SAA and Hp in the liver [49]. Certain cell membrane proteins, TLR-4 and CD14, join with LPS and then attach to TRIF, which is an adapter-inducing interferon-β. Consequently, the activation of macrophages in the signalling pathway leads to the release of inflammatory cytokines, including IL-1 and IL-6. These inflammatory cytokines are released to promote blood circulation and induce the liver to synthesise APPs [48].

In this study, the selected parameters of the immune system in cows with I-NEFA (NEFA 0.24 ± 0.12) and II-NEFA class (NEFA 0.87 ± 0.23) groups were thoroughly assessed. This investigation identified significant differences in serum SAA between the I-NEFA class and the II-NEFA class. This research found a 24.19% reduction in the levels of SAA in the bloodstream in the II-NEFA class compared to the I-NEFA class. Additionally, this research identified a substantial negative correlation between blood NEFAs and SAA (r = −0.441, *p* < 0.001). These findings suggest that there may be a correlation between NEFA levels and SAA in cows. A recent study revealed that cows with clinical ketosis exhibited less pronounced inflammation compared to those with subclinical ketosis. The indices in cows with clinical ketosis blood SAA were comparable to those in healthy cows (control group) and substantially lower than those in cows with subclinical ketosis [50]. Similar results were obtained in our study, when blood SAA was lower in II-NEFA class than in I-NEFA class. SAA is an apolipoprotein, which appears in the bloodstream as the first response protein up to 24–48 h after the occurrence of an inflammatory factor, such as infection, and its secretion is dependent on IL-1 and/or TNF-α. This may be a reason that in cows with subclinical ketosis, inflammation occurs first, which is why the increased SAA concentrations are noted; however, this condition may last for a different time period before it is diagnosed, and for that reason, Hp concentrations also increase [50]. This represents an innovative perspective on the disease entity, as most publications regard the released cytokines and APP as diagnostic markers for ketosis [51]. Nonetheless, to our knowledge, these indicators lack specificity, and their levels also elevate in several other disorders [52]. This study, along with the findings of Brdzki et al., does not allow for a definitive conclusion regarding whether ketosis or elevated blood levels of NEFAs in dairy cows are preceded by systemic inflammation, nor is it clear if inflammation influences the development of ketosis [50]. Inflammation may contribute to the onset of ketosis; however, further research is required to validate this notion. Furthermore, pinpointing the distinct markers exclusive to ketosis may enhance diagnostic precision and therapeutic approaches for dairy cows afflicted by this illness. These data indicate a complex interaction among inflammation, NEFA levels, and ketosis that requires additional research.

## 5. Conclusions

This study investigated the associations among blood NEFAs, SAA, milk composition, and the behavioural parameters of fresh dairy cows. The findings revealed a substantial negative correlation between blood NEFAs and SAA. As NEFA levels rise, blood SAA levels decrease, indicating that increased lipomobilisation from body reserves may suppress the acute-phase protein response. The F:P in milk was 13.67% higher in the II-NEFA class (NEFA 0.87 ± 0.23) compared to the I-NEFA class (NEFA 0.24 ± 0.12). That indicates that elevated NEFA levels are indicative of energy mobilisation from body reserves, which is commonly observed during energy deficits or metabolic stress. Additionally, there is a notable inverse association between SAA and the milk F:P, indicating that inflammation may negatively affect milk fat synthesis. Increased SAA levels were linked to elevated cow activity, suggesting behavioural changes driven by pain or discomfort caused by inflammation. Changes in the milk F:P ratio serve as critical indicators of the metabolic status of dairy cows, reflecting their energy balance and overall health during lactation.

Dairy farmers have the opportunity to detect SARA early by closely monitoring the milk F:P and making timely adjustments to feeding practices. This proactive approach can significantly improve the overall health and productivity of the cows, while optimizing milk production efficiency. Moreover, comprehending the correlation between the milk content and metabolic processes may yield significant insights for formulating targeted feeding strategies customised to the specific requirements of cows.

Further research is needed to fully understand the mechanisms behind these relationships and how they may impact milk production and cow health in the long term. In addition to exploring the effects of inflammation on milk composition, future studies could also investigate potential interventions or management strategies to mitigate the negative impact of elevated NEFA and SAA levels on dairy cow performance. By gaining a better understanding of these relationships, dairy farmers and veterinarians may be able to improve the overall health and productivity of their herds.

## Figures and Tables

**Table 1:** Chemical composition of dietary feeds for lactating dairy cows.

Parameters	Units	Dairy Cows
Dry matter	%	44.0
Dry matter intake	kg DM/d	26.5
Net energy lactation	MJ/kg DM	7.01
Crude protein	g/kg DM	175
Crude fat	g/kg DM	45
Fatty acids	g/kg DM	34
Protein balance in rumen	g/kg DM	22
Neutral detergent fiber	g/kg DM	287
Starch	g/kg DM	204

kg DM/d—kilograms of dry matter per day; MJ/kg DM—megajoules per kilogram of dry matter; g/kg DM—grams per kilogram of dry matter.

**Table 2:** Correlation among blood, milk, and behaviour parameters.

Correlations									
Indices		NEFAs mmol/L	SAA μg/mL	Milk Fat	Milk Protein	Milk F:P	Reticulorumen pH	Rumination Time min./d.	Activity h/day
NEFAs mmol/L	r	1							
	P								
	N	71							
SAA μg/mL	Pearson correlation	−0.441 **	1						
	Sig. (2-tailed)	0.001							
	N	71	71						
Milk fat	Pearson correlation	0.459 **	−0.426 **	1					
	Sig. (2-tailed)	0.001	0.001						
	N	71	71	71					
Milk protein	Pearson correlation	−0.179	0.293 *	0.053	1				
	Sig. (2-tailed)	0.135	0.013	0.659					
	N	71	71	71	71				
Milk F:P	Pearson correlation	0.516 **	−0.535 **	0.884 **	−0.413 **	1			
	Sig. (2-tailed)	0.001	0.001	0.000	0.000				
	N	71	71	71	71	71			
Reticulorumen pH	Pearson correlation	0.204	−0.027	0.233	−0.044	0.225	1		
	Sig. (2-tailed)	0.088	0.826	0.051	0.717	0.059			
	N	71	71	71	71	71	71		
Rumination time min./d.	Pearson correlation	−0.008	−0.139	0.100	0.008	0.091	0.416 **	1	
	Sig. (2-tailed)	0.949	0.249	0.408	0.946	0.448	0.001		
	N	71	71	71	71	71	71	71	
Activity h/day	Pearson correlation	−0.279 *	0.382 **	−0.389 **	0.047	−0.389 **	0.021	−0.223	1
	Sig. (2-tailed)	0.019	0.001	0.001	0.695	0.001	0.862	0.061	
	N	71	71	71	71	71	71	71	71

** Correlation r (Pearson’s correlation coefficient) is significant at *p* < 0.01; * Correlation is significant *p* < 0.05; NEFAs—nonesterified fatty acids; SAA—serum amyloid A; milk F:P—milk fat-to-protein ratio.

**Table 3:** Descriptive statistics of the milk parameters.

Descriptives									
Parameter	NEFA Class	N	Mean	Std. Deviation	95% Confidence Interval for Mean		Minimum	Maximum	*p*
					Lower Bound	Upper Bound			
Milk fat	0.24 ± 0.12	43	3.81	0.58	3.63	3.99	2.64	5.32	0.01
	0.87 ± 0.23	28	4.20	0.71	3.93	4.47	2.98	5.52	
	Total	71	3.97	0.66	3.81	4.12	2.64	5.52	
Milk protein	0.24 ± 0.12	43	3.28	0.25	3.20	3.36	2.66	3.95	0.11
	0.87 ± 0.23	28	3.18	0.29	3.06	3.29	2.64	3.82	
	Total	71	3.24	0.27	3.18	3.30	2.64	3.95	
Milk fat-to-protein ratio	0.24 ± 0.12	43	1.17	0.18	1.11	1.22	0.79	1.64	<0.001
	0.87 ± 0.23	28	1.33	0.26	1.23	1.43	0.88	1.95	
	Total	71	1.23	0.23	1.18	1.29	0.79	1.95	

**Table 4:** Descriptive statistics of the blood parameters.

Descriptives									
Parameter	NEFA Class	N	Mean	Std. Deviation	95% Confidence Interval for Mean		Minimum	Maximum	*p*
					Lower Bound	Upper Bound			
SAA (μg/mL)	0.24 ± 0.12	43	77.84	23.38	70.65	85.04	23.81	100.00	<0.001
	0.87 ± 0.23	28	59.01	20.99	50.88	67.15	23.81	100.00	
	Total	71	70.42	24.16	64.70	76.14	23.81	100.00	

## Data Availability

The study’s original contributions are contained within the journal; further enquiries may be directed to the corresponding author.
